# P63 modulates the expression of the *WDFY2* gene which is implicated in cancer regulation and limb development

**DOI:** 10.1042/BSR20192114

**Published:** 2019-12-13

**Authors:** Paola Monti, Yari Ciribilli, Giorgia Foggetti, Paola Menichini, Alessandra Bisio, Serena Cappato, Alberto Inga, Maria Teresa Divizia, Margherita Lerone, Renata Bocciardi, Gilberto Fronza

**Affiliations:** 1 Mutagenesis and Cancer Prevention Unit, IRCCS Ospedale Policlinico San Martino, Largo R. Benzi, 10, Genoa 16132, Italy; 2 Department of Cellular, Computational and Integrative Biology (CIBio), University of Trento, Via Sommarive 9, Povo (TN) 38123, Italy; 3 Department of Neurosciences, Rehabilitation, Ophthalmology, Genetics, Maternal and Child Health (DiNOGMI), University of Genoa, Largo P. Daneo, 3, Genoa 16132, Italy; 4 Medical Genetics Unit, IRCCS Istituto Giannina Gaslini, Via G. Gaslini, 5, Genoa 16147, Italy

**Keywords:** cancer regulation, functional analysis, limb development, P63, target gene, WDFY2

## Abstract

*TP63* is a member of the *TP53* gene family, sharing a common gene structure that produces two groups of mRNAs’ encoding proteins with different N-terminal regions (ΔN and TA isoforms); both transcripts are also subjected to alternative splicing mechanisms at C-terminus, generating a variety of isoforms. p63 is a master regulator of epidermal development and homoeostasis as well as an important player in tumorigenesis and cancer progression with both oncogenic and tumour suppressive roles. A number of studies have aimed at the identification of p63 target genes, allowing the dissection of the molecular pathways orchestrated by the different isoforms. In the present study we investigated in more detail the p63 responsiveness of the *WDFY2* (*WD* repeat and *FY*VE domain containing *2*) gene, encoding for an endosomal protein identified as a binding partner of the PI-3K/AKT signalling pathway. We showed that overexpression of different p63 isoforms was able to induce *WDFY2* expression in *TP53*-null cells. The p63-dependent transcriptional activation was associated with specific response elements (REs) that have been identified by a bioinformatics tool and validated by yeast- and mammal-based assays. Interestingly, to confirm that *WDFY2* belongs to the p63 network of cancer regulation, we analysed the impact of *WDFY2* alterations, by showing its frequent deletion in different types of tumours and suggesting its expression level as a prognostic biomarker. Lastly, we identified a chromosomal translocation involving the *WDFY2* locus in a patient affected by a rare congenital limb anomaly, indicating *WDFY2* as a possible susceptibility gene placed downstream p63 in the network of limb development.

## Introduction

*TP63*, a member of the *TP53* gene family [1], encodes a sequence-specific transcription factor able to modulate the expression of genes involved in different pathways such as development and tumorigenesis [2–4]. The modulation of these processes depends on the expression of different N-terminal (ΔN and TA) as well as C-terminal (α, β, γ, δ and ε) isoforms deriving from either the use of alternative promoters (P1 and P2) or splicing sites [5].

ΔNp63α is the most highly expressed p63 isoform in the basal layer of epithelial tissues where it plays a crucial role in their proliferation and terminal differentiation; conversely, TAp63 isoforms are expressed in response to DNA damage in the epithelium and in the germline cells, where they control genomic stability and integrity. Accordingly, ΔNp63 knockout mice exhibit severe developmental defects, including limbs truncation and the failure in developing mature stratified epithelia [6], while TAp63 knockout mice develop blisters, ulcerated wounds and age prematurely [7].

In humans, germline heterozygous mutations in the *TP63* gene, mainly affecting at protein level ΔNp63α, underlie the molecular basis of a subset of ectodermal dysplasia syndromes whose clinical features clearly correlate with the phenotype of the ΔNp63 knockout mice. In fact, these clinical conditions share three main characteristics in various combinations: ectodermal dysplasia, split hand/foot malformation and orofacial clefting [8].

With regard to the role of p63 in cancer, early genetic studies in mice reported conflicting results. Flores and colleagues [9] observed the development of spontaneous tumours in *TP63*^+/−^ mice, while Keyes and Mills [10] did not obtain the same results, this difference being probably due to the failure to create p63 isoform-specific knockout mice. In fact, the ability of TAp63 isoforms to induce apoptosis, cell cycle arrest and senescence is fundamental for the tumour suppression function; accordingly, TAp63 heterozygous and null mice develop spontaneous carcinomas and sarcomas [11]. Conversely, the capacity of ΔNp63 isoforms to interfere with p53-, p63- and p73-dependent transcription seems to be the basis of their oncogenic role. Moreover, ΔNp63 isoforms may contribute directly to malignant transformation by transcriptionally regulating the expression of some pro-survival genes [12,13]. To further complicate the scenario in cancer, the unbalanced co-expression of each individual p63 isoform, as well as the interaction with other p53 family members, including mutant p53, is a frequent event [14].

p63 regulation of target genes is accomplished mainly by the binding of p63 to response elements (REs) similar to those of p53 (RRRCWWGYYY-n-RRRCWWGYYY; R = purine, W = A or T, Y = pyrimidine, n = 0–13 bps spacer; CWWG = core sequence) [15]. However, Perez and colleagues [16] by using Systematic Evolution of Ligands by EXponential enrichment (SELEX) technique highlighted a degeneration of the p63 consensus motif with respect to the p53 RE, partially explaining the ability of p63 to activate the transcription from REs distinct from those activated by p53. We also demonstrated that TAp63α and ΔNp63α proteins, tested against a panel of p53 REs, exhibited differences in transactivation specificity not appreciable for the corresponding p53 and p73 isoforms [17]. In addition, the chromatin architecture and the cooperation of other transcription factors or cofactors can modulate p63 activity [18].

Previously, Vigano and colleagues [19] identified *WDFY2* (*WD* repeat and *FY*VE domain containing *2*) as a putative p63 target gene through Chromatin ImmunoPrecipitation (ChIP) on-chip experiments in human immortalised HaCaT keratinocytes. The human *WDFY2* gene, located on chromosome 13 at 13q14.3 locus, encodes a well-conserved protein of 400 residues that is characterised by the presence of seven WD40 motifs and a FYVE domain [20]. WD40 motif consists of a module of approximately 40 residues working as protein–protein or protein–DNA interaction platform, while the FYVE domain is known to bind Phosphatidyl-Inositol 3-Phosphate (PI3P), a major product of PI3K and found almost exclusively on the surface of endosomes. WDFY2 was indeed characterised in *Caenorhabditis elegans* in a study aimed at identifying proteins able to bind PI3P and involved in endocytosis [21]. Although the function of *WDFY2* gene has been poorly defined, some pieces of evidence are recently linking WDFY2 protein to PI3K/AKT signalling transduction pathway that play a key role in cancer tumour progression [22].

Little is known about the transcriptional regulation of the *WDFY2* gene; in the present study we investigated in more details the responsiveness of *WDFY2* gene to the p63 transcription factor, confirming *WDFY2* as a putative target with implications for the p63 activity as a tumour suppressor and as a regulator of limb development. Interestingly, *WDFY2* expression appeared to be also modulated by the tumour suppressor p53 protein.

## Materials and methods

### *In silico* analysis of human *WDFY2* genomic organisation

The genomic organisation of human *WDFY2* gene was retrieved from UCSC Genome database (http://genome.ucsc.edu/ GRCh38/hg38 Assembly; RefSeq NM_052950). The TFBIND Software that uses positional weight matrices from transcription factor database TRANSFAC R.3.4 (http://tfbind.hgc.jp/) [23] was applied; the search of the REs was focused on the promoter and intron 1 regions [10 kb upstream and 20 kb downstream of the transcription start site (TSS), respectively] according to the study by Vigano and colleagues [19], in which *WDFY2* locus was found strongly enriched for p63 binding in these regions. Amongst the binding sites retrieved by TFBIND Software, we selected canonical p53 binding sites (20 bps without a spacer).

### Yeast strains and mammalian cell lines

A panel of yLFM-WDFY2 yeast strains was constructed using the delitto perfetto approach [24]. The yIG397 strain was used for the Gap Repair Assay [25]. Yeast manipulations were performed as previously described [26]. HCT116 *TP53^−/−^* cells (human colon carcinoma) were obtained by Dr. B. Vogelstein (The Johns Hopkins Kimmel Cancer Center, Baltimore, MD) and grown as previously described [27].

### Yeast and mammalian vectors

p53, p63 (ΔNp63α, ΔNp63β, TAp63α and TAp63β) and p73 (ΔNp73α, ΔNp73β, TAp73α and TAp73β) proteins were expressed by an available yeast pTSG-based vector (TRP1) [26,27]. The yeast plasmids encoding the ΔNp63γ and TAp63γ isoforms were constructed starting from available mammalian pCDNA3 expression vectors (generous gift from Dr. E. Candi (University of Rome “Tor Vergata”, Rome, Italy; IRCCS, Rome, Italy), Supplementary Materials and Methods) as previously described [26]. Plasmid pRS314 was used as an empty vector.

Mammalian pCI-neo plasmids for the expression of ΔNp63α, ΔNp63β, TAp63α and TAp63β were already available [26]. pCI-neo plasmids expressing ΔNp63γ and TAp63γ isoforms were obtained as previously described [17,26]. Plasmid pCI-neo was used as an empty vector.

For the construction of the mammalian reporter vectors, a genomic fragment (approximately 3 kb) upstream of the *WDFY2* TSS and containing the predicted REs was amplified by PCR with the AmpliTaq Gold DNA Polymerase (Thermo Fisher Scientific, Walthman, MA, U.S.A.) by using the oligonucleotides indicated in Supplementary Table S1. The obtained product was cloned into the pCR2.1 vector from the TOPO-TA cloning kit (Thermo Fisher Scientific) and checked by sequencing. The insert was isolated from the TA vector clones by KpnI-XhoI (NEB, Ipswich, Massachusetts, U.S.A.) digestion and then cloned into the corresponding sites of the pGL4.17 reporter vector (Promega, Madison, WI, U.S.A.) upstream of the luciferase coding sequence (reporter vector indicated as Pr3300). The +12.5 RE was cloned into the BamHI site of the Pr3300 reporter vector (sense and antisense) (reporter vector indicated as Pr3300+12.5 RE S and Pr3300+12.5 RE aS) by using the oligonucleotides indicated in Supplementary Table S1. Deletion of the −3.3 and −0.5 REs in the Pr3300 reporter vector (reporter vector indicated as Pr3300 del−3.3/−0.5 REs) was obtained by site-directed mutagenesis using the QuikChange Lightning Site-Directed Mutagenesis Kit (Agilent Technologies, Santa Clara, CA, U.S.A.) with the oligonucleotides indicated in Supplementary Table S1.

### Western blot analysis

Yeast or mammalian protein extraction and immunodetection were performed as previously described [26–28] by loading 10 μl of yeast supernatant or 20–50 μg of mammalian cell lysates. Specific antibodies directed against human p63 (clone 4A4, obtained either from Oncogene Research Products, La Jolla, CA, U.S.A., and Ab1, Santa Cruz Biotechnology, Dallas, TX, U.S.A.), yeast PGK1 (Phospho Glycerate Kinase 1, clone 22C5D8, Life Technologies), human p53 (clone DO-1, Santa Cruz Biotechnology), human WDFY2 (ab176895, Abcam, Cambridge, U.K.), human α-Actinin (clone H-2, Santa Cruz Biotechnology), human β-Actin (clone AC-74, Sigma–Aldrich) and the appropriate IgG-horseradish peroxidase–conjugated secondary antibody (anti-mouse or anti-rabbit IgG HRP, Sigma–Aldrich) were used.

### Reporter assays

The yeast functional assay was conducted according to the miniaturised protocol we developed [26,27,29]. The mammalian reporter assay was performed in HCT116 *TP53*^−/−^ cells as previously described [26,30]. The transactivation ability of the proteins was expressed as fold over the empty vector (pRS314 or pCI-neo).

### Endogenous gene expression analysis

HCT116 *TP53*^−/−^ cells were seeded in six-well plates (0.9 × 10^6^ cells) and transfected with 2 μg of the different pCI-neo expression vectors using the Lipofectamine LTX transfection reagent (Life Technologies) according to the manufacturer’s instructions. Total RNA was extracted using Illustra RNA spin Mini Kit (GE Healthcare, Euroclone) and converted into cDNA using the RevertAid First Strand cDNA Synthesis kit following manufacturer’s recommendations (Thermo Fisher Scientific). Endogenous *WDFY2* levels were analysed using KAPA Sybr Fast qPCR Master Mix (KAPA Biosystem, Sigma–Aldrich) and measured with a CFX384 Detection System (Bio-Rad). Relative expression levels were calculated as fold over the empty vector (pCI-neo) for each expression plasmid using the ΔΔ*C*_t_ method with the *GAPDH* and *YWHAZ* reference genes, as previously described [31]. Primers (Supplementary Table S1) were picked using Primer-BLAST designing tool (https://www.ncbi.nlm.nih.gov/tools/primerblast/) and controlled for specificity and for efficiency with a standard curve.

### Statistical analyses

Statistical analyses were performed using a two-tailed unpaired *t* test; a *P*-value lower than 0.05 was considered to be statistically significant. Main statistical results are reported in the figures and/or text; we indicated a *P*-value ≤ when we reported the highest *P*-value. Results of exhaustive statistical analyses are shown in Supplementary Table S2. The statistical calculations were performed with GraphPad Prism 6.0 for Windows (GraphPad Software, La Jolla, CA, U.S.A.).

### *WDFY2* genome-wide analysis in cancer

The genetic events involving *WDFY2* gene in cancer samples were determined by using the cBioPortal online tool for cancer genomics that is developed by The Cancer Genome Atlas consortium (TCGA, http://www.cbioportal.org/) [32,33]. To enforce our analysis, the studies with at least 1,000 patients were selected.

To evaluate the clinical significance of *WDFY2* expression levels, a panel of gene expression datasets from cancer patients was analysed. We selected the cancer types where Illumina RNA-seq experiments from TCGA consortium (https://cancergenome.nih.gov/) were available, comprising datasets from cancer, as well as normal tissues, and involving more than 30 samples. Gene expression data (RPKM, reads per kilobase per million mapped reads) were obtained from Synapse online tool, as previously described [34] (https://www.synapse.org/; TCGA_Pancancer 12; [35]) on lung adenocarcinomas (LUAD) {Synapse ID: syn418003; [36]}, breast invasive carcinomas (BRCA) {Synapse ID: syn1446183; [37]}, renal clear cell carcinomas (RCC) {Synapse ID: syn1446226; [38]}, and head and neck squamous cell carcinomas (HNSCC) {Synapse ID: syn1461156; [39]}. Moreover, RNA-seq data from 42 Oestrogen Receptor positive (ER+) and 42 Triple-negative breast cancer (TNBC) patients as well as their matched unaffected adjacent tissues (30 and 21 samples, respectively) were analysed {GSE58135 [40]}. *WDFY2* expression levels in cancer were also evaluated using gene expression profiling interactive analysis (GEPIA) tool [41].

To evaluate the prognostic value of *WDFY2* expression, Kaplan–Meier survival curves were generated for breast cancer (1764 patients) [42] or LUAD patients (673 patients) [43] using the Kaplan–Meier plotter online tool (http://kmplot.com/analysis/). The Luminal A (841), Luminal B (407), HER2+ (156) and Basal (360) subgroups according to PAM50 intrinsic classification were also interrogated separately [44]. Patients were divided for *WDFY2* expression; hazard ratios (HRs) and *P*-values were generated for statistical significance according to the Relapse-Free Survival (RFS) across 25 years of follow-up.

### Cytogenetic and molecular analyses

Chromosome analysis was performed by standard cytogenetic techniques (Q-banding) on the blood lymphocytes after informed consent from the patients (Supplementary Materials and Methods).

The rearrangement breakpoints were refined at the BAC level by FISH analysis as described in Bocciardi et al. [45]. BAC clones selected from the human library RPCI-11 (UCSC Human Genome Assembly, http://genome.ucsc.edu/cgi-bin/hg.Gateway; May 2006) were obtained from the YAC Screening Center (DIBIT, Milan) and probed for FISH (Supplementary Table S3). This analysis allowed us to exclude the presence of any additional micro-rearrangements (i.e., microdeletions or duplications) in the regions immediately surrounding the breakpoints.

CGH-Array Analysis of genomic DNA was performed from whole blood with the Agilent Human Genome CGH Microarray Kit 44B (average resolution 75 kb, Agilent Technologies) as described in Tassano et al. [46]. Breakpoint spanning sequences were analysed by the bioinformatic tools at NCBI (www.ncbi.nlm.nih.gov; Blast and Map viewer) and UCSC Genome browser (http://genome.ucsc.edu). Screening of the 16 coding exons of the *TP63* gene was performed as previously described [47].

## Results

### p63 is able to transactivate from some REs identified in the human *WDFY2* gene

Although Vigano and colleagues [19] identified *WDFY2* amongst the genes induced by p63, a systematic search and validation of the REs involved in its regulation have never been carried out. By applying the TFBIND software, we identified seven putative REs, four located in the genomic region upstream of the TSS, and three in the first intron (Table 1). In order to study their functionality, seven yeast strains were constructed; the strains are isogenic except for the different *WDFY2* RE located upstream from the luciferase reporter gene. Since the REs were identified as p53 REs, the transactivation ability of all p53 family members was verified.

**Table 1:** REs in human *WDFY2* gene

Name	Location (bp from TSS) GRCh38/hg38 assembly	Sequence (PuPuPuC(A/T)(A/T)GPyPyPy) (PuPuPuC(A/T)(A/T)GPyPyPy)
−7.6	Promoter (−7,619)	5′-AAACAAATCT-GGACCTGCCT-3′
−4.3	Promoter (−4,340)	5′-AGGCATGCGC-AACCAAGCCC-3′
−3.3	Promoter (−3,302)	5′-GAGCTTGTCC-ACACCTGTCC-3′
−0.5	Promoter (−537)	5′-GCGCATGCCA-AGTCACGTCC-3′
+12.5	Intron 1 (+12,507)	5′-GGGCATGTGT-GGGCTTGTCT-3′
+14.5	Intron 1 (+14,543)	5′-AGGCATGCGC-TACCATGCCC-3′
+15.7	Intron 1 (+15,785)	5′-AAACCAGCTC-TGGCATTTCC-3′

The nucleotides underlined represent mismatches with respect to the p53 consensus sequence.

**Figure 1:**
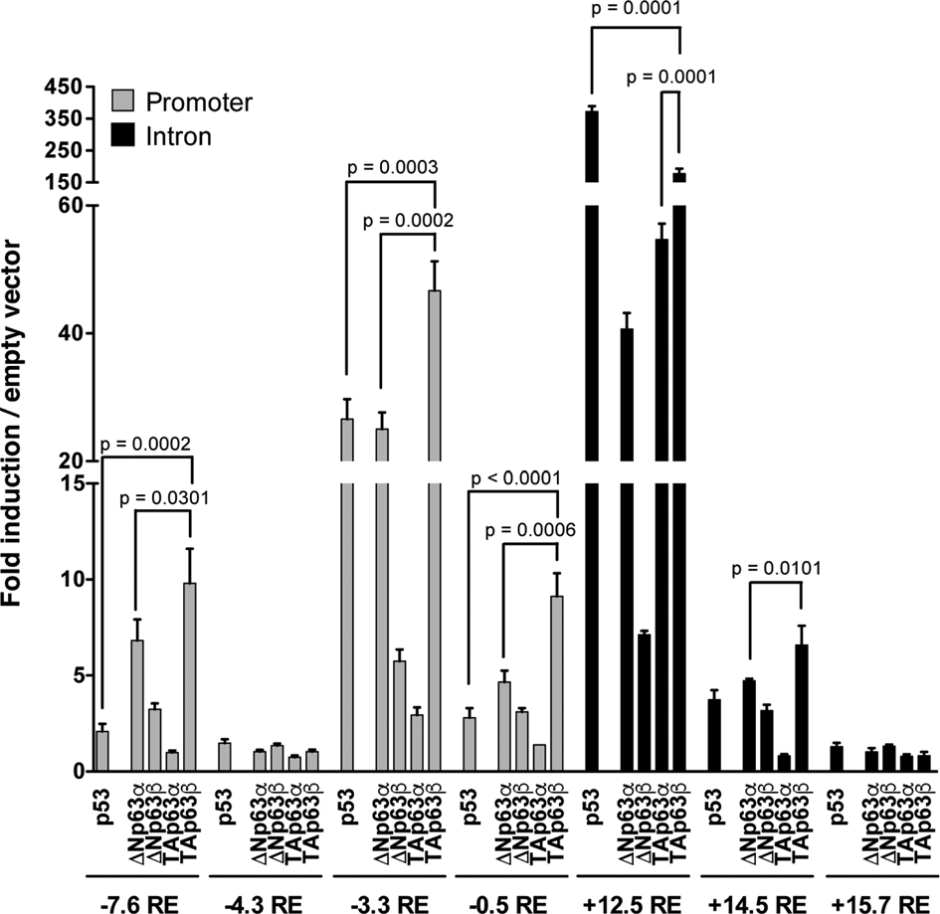
P63 isoforms transactivation from REs belonging to *WDFY2* gene by using a yeast-based reporter assay Transactivation ability of p53 and p63 isoforms in yLFM-WDFY2 yeast strains containing the reporter gene under the regulation of a promoter (−7.6, −4.3, −3.3 and −0.5) or intron 1 (+12.5, +14.5 and +15.7) RE from the human *WDFY2* gene. The transactivation ability was determined using an inducible expression of the proteins (*GAL1,10* promoter) and by growing yeast cells for 8 h in media containing 0.128% Galactose. Presented is the fold of induction over the empty vector and the standard deviation of four biological replicates.

Regarding the REs located in the *WDFY2* promoter (−7.6, −4.3, −3.3 and −0.5), p63 isoforms are able to transactivate to variable extent, being the yeast strain with the −3.3 RE the most responsive (Figure 1). Very weak transactivation by p63 isoforms is detectable from the −4.3 RE. Amongst the p63 isoforms, TAp63β shows the highest transactivation ability (*P*≤0.0301); moreover, ΔNp63α appears to be always more active than ΔNp63β and TAp63α (*P*≤0.0026). On the other hand, p53 is less active than TAp63β (*P*≤0.0003) and all p73 isoforms show very weak transactivation ability (Supplementary Figure S1A).

Considering the REs identified in intron 1 (+12.5, +14.5 and +15.7), p63 isoforms show high activity in yLFM-WDFY2 +12.5 RE, weak transactivation in yLFM-WDFY2 +14.5 RE, and almost no transactivation in yLFM-WDFY2 +15.7 RE (Figure 1), being TAp63β the most active isoform (*P*≤0.0101). p53 is a more potent transcription factor than all p63 isoforms only in the yeast strain that contain the +12.5 RE (*P*<0.0001), where also TAp73α and TAp73β show some transactivation ability, even though lower than the corresponding TAp63 isoforms (*P*<0.0001) (Supplementary Figure S1A). Taken collectively, these data indicated that the REs identified in the human *WDFY2* gene are more efficiently transactivated by p63 than by p73, and, in most cases, also than by p53.

Since it is known that the TAp63γ is the most potent transcription factor within p63 isoforms [1,48], the γ isoforms (ΔN and TA) were cloned and tested by using the most responsive yLFM-WDFY2 strains (i.e. −3.3 and +12.5) and two different galactose concentrations to modulate the level of protein expression [49]. The results show that the TAp63γ protein has the highest transactivation ability, especially appreciable at low expression level (*P*<0.0001 at 0.016% Galactose in yLFM-WDFY2 +12.5 RE, Supplementary Figure S1B), while the ΔNp63γ isoform is weakly active with respect to almost all p63 isoforms, even at higher expression level (0.128% Galactose, Supplementary Figure S1B). By comparing the transactivation activity of the p63 isoforms with their respective protein levels (Supplementary Figure S1C), we can conclude that: (1) the −3.3 and +12.5 *WDFY2* REs are efficiently transactivated by the less expressed TAp63β and TAp63γ isoforms and (2) amongst ΔNp63 isoforms, ΔNp63α protein shows substantial transactivation activity on both *WDFY2* REs.

### p63 isoforms expressed in human cells transcriptionally activate the *WDFY2* promoter

The identification of functional REs in *WDFY2* gene prompted us to test the transcriptional activity of the different p63 isoforms in human cells. To this aim, a portion of the human *WDFY2* promoter containing the −3.3 RE (i.e., the most responsive in yeast assay) and the −0.5 RE (i.e., the most proximal to the TSS) was cloned in a mammalian reporter vector. To determine the role of the identified REs in the p63-dependent regulation of *WDFY2*, a promoter with deletion of both REs was also constructed. The reporter assay was performed in human colon cancer HCT116 *TP53*^−/−^ cells, in which p63 isoforms were not detectable [26].

**Figure 2:**
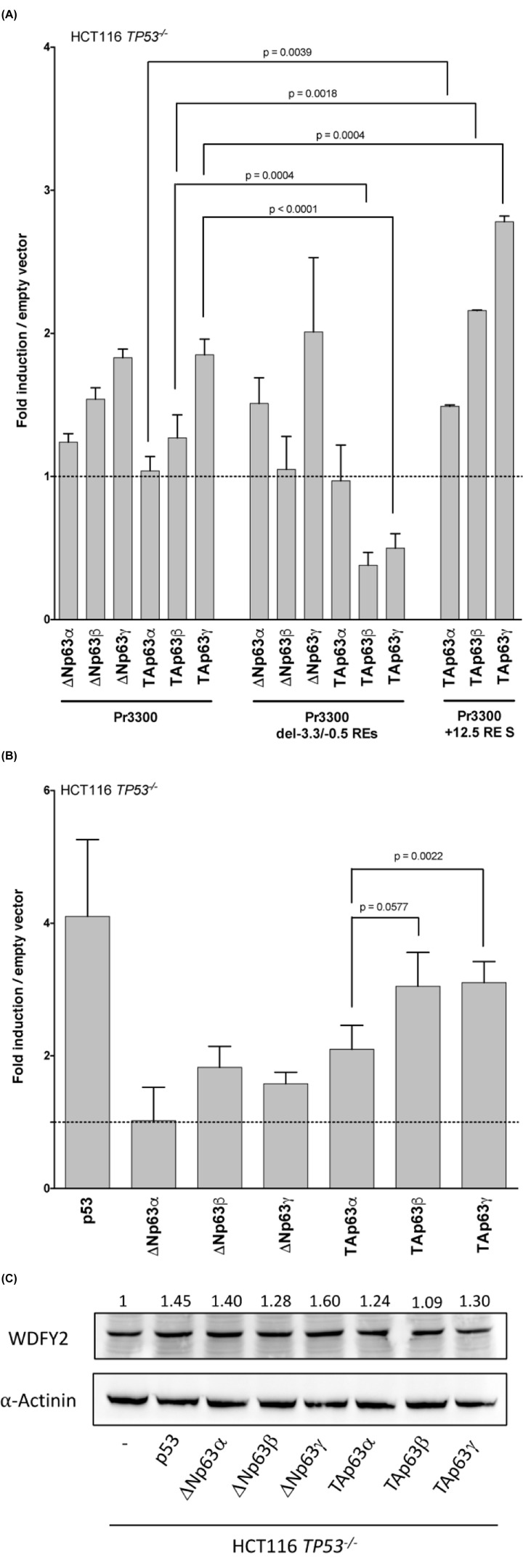
Regulation of WDFY2 expression by P63 isoforms in human cells (**A**) Transactivation ability of p63 isoforms on reporter constructs (Pr3300, Prom3300 del−3.3/−0.5 REs, Pr3300 +12.5 RE S) in HCT116 *TP53*^−/−^ human cells. *Renilla* luciferase was used to normalise for transfection efficiencies. Data are expressed as fold of induction relative to the results obtained with the empty vector. Presented are the average and standard deviations of at least three independent experiments performed in triplicate. Fold of induction of TAp63α, TAp63β and TAp63γ on Pr3300 +12.5 RE aS (1.31, 1.91 and 2.59, respectively) (data not shown) are slightly lower but comparable with the corresponding values on Pr3300 +12.5 RE S (1.49, 2.16 and 2.78, respectively). (**B**) Endogenous mRNA expression level of the *WDFY2* target in HCT116 *TP53*^−/−^ cells transfected with the p53 and p63 expression plasmids. Relative expression levels of *WDFY2* were calculated respective to *GAPDH* and *YWHAZ* reference genes. Bars represent the averages and standard deviations of at least three independent experiments. (**C**) Representative Western blot showing the level of WDFY2 protein found in HCT116 *TP53*^−/−^ cell lysates following transient transfection with the indicated p63 and p53 expression vectors. α-Actinin was used for normalisation.

p63 isoforms are able to activate *WDFY2* promoter (Pr3300) to a variable extent; in the absence of −3.3 and −0.5 REs (Pr3300 del−3.3/−0.5 REs) TAp63β and TAp63γ isoforms clearly lose their transactivation (*P*=0.0004 and *P*<0.0001, respectively) (Figure 2A); furthermore, ΔNp63β shows a slight decrease in the transactivation ability (*P*=0.0098). To correlate the transactivation activity of p63 isoforms with their protein level, cell extracts were analysed by Western Blot (Supplementary Figure S2A). Consistent with data in the literature [48], TAp63β and TAp63γ isoforms are expressed at low levels in human cells after transfection, suggesting that their transactivation ability can be underestimated. Taken together, these results suggest that the TAp63β and TAp63γ variants are the most potent activators of *WDFY2* promoter construct; moreover, it emerges that also the ΔNp63α and ΔNp63β isoforms are able to function as transcription factors on the promoter.

The functional +12.5 RE, identified in intron 1 of *WDFY2*, was also tested as an enhancer element against the most transactivating TAp63β and TAp63γ isoforms and the less active TAp63α, by cloning it into the *WDFY2* promoter vector (Pr3300 +12.5 RE S) (Figure 2A). The results show that the presence of the +12.5 RE produces a slightly higher transactivation by all TAp63 isoforms with respect to the activity observed with the promoter without the enhancer RE (*P*=0.0039, *P*=0.0018 and *P*=0.0004, respectively).

Lastly, the *WDFY2* promoter was tested with p53 in the same human cell line; the results indicate that also p53 is able to transactivate the *WDFY2* promoter, a feature lost when the −3.3 and −0.5 REs are absent (*P*<0.0001) (Supplementary Figure S2 B,C).

### p63 is able to regulate the endogenous *WDFY2* gene

To determine if p63 isoforms were capable of driving the expression of the endogenous *WDFY2* gene in mammalian cells, p63 isoforms were transiently expressed into HCT116 *TP*53^−/−^ cells.

Compared with the empty vector, all TAp63 isoforms show more than two-fold induction of *WDFY2* mRNA, with TAp63β and TAp63γ tending to be the most active (Figure 2B) but the less expressed as previously observed; moreover, ΔNp63β and ΔNp63γ display residual induction ability (Figure 2B). The results indicate the general capability of the p63 isoforms to induce the expression of the endogenous *WDFY2* gene; in addition, also p53 is able to modulate its mRNA level (Figure 2B). Lastly, following transient transfection of HCT116 *TP*53^−/−^ cells with p63 isoforms or p53, the level of WDFY2 protein slightly increases (Figure 2C).

### Placing *WDFY2* into the p63 network of tumour regulation: impact of *WDFY2* alterations in cancer

By considering *WDFY2* as a p63 effector, potentially involved in cancer regulation, we investigated *WDFY2* alterations in tumours.

The genetic events involving *WDFY2* (71,088 samples from a panel of 234 different cancer studies) comprise deep deletions, mutations, amplifications and fusions. *WDFY2* results altered in 842 (1%) of all samples. The large majority of the *WDFY2* mutations are missense mutations (136/150) spread all over the sequence of the gene (Supplementary Figure S3A).

*WDFY2* appears to be deleted in many cancer types from different origins (blue columns in Supplementary Figure S3B,C), with the highest frequency in prostate (>5% in most of the studies) and bladder cancers (3–4% of the cases by selecting the individual studies with more than 100 patients). Conversely, despite being rare in general, mutations (green columns) were found more often in lung, melanoma, uterus and pancreas (Supplementary Figure S3B,C). *WDFY2* amplifications (red columns), also generally rare, were relatively more frequent in bowel, oesophagus/stomach and kidney cancer samples (Supplementary Figure S3C).

We then evaluated *WDFY2* expression in human cancers (i.e., lung, breast, kidney and head and neck) (Supplementary Figure S4A–D). Interestingly, significantly lower levels of *WDFY2* are observed in cancer tissues with respect to matched normal tissues with the exception of HNSCC, where no difference is present. We also analysed an additional RNA-seq dataset from different subtypes of breast cancers (ER+ and TNBCs) in comparison with the adjacent normal tissues, highlighting a significant reduction in *WDFY2* in ER+ but not in TNBCs (Supplementary Figure S4E). An extended comparison done with the GEPIA online tool [41] confirmed that *WDFY2* expression is significantly reduced in different cancer types (acronyms in green font) with the only exception of Acute Myeloid Leukemia (LAML, red font) where an opposite effect is visible (Supplementary Figure S4F).

**Figure 3:**
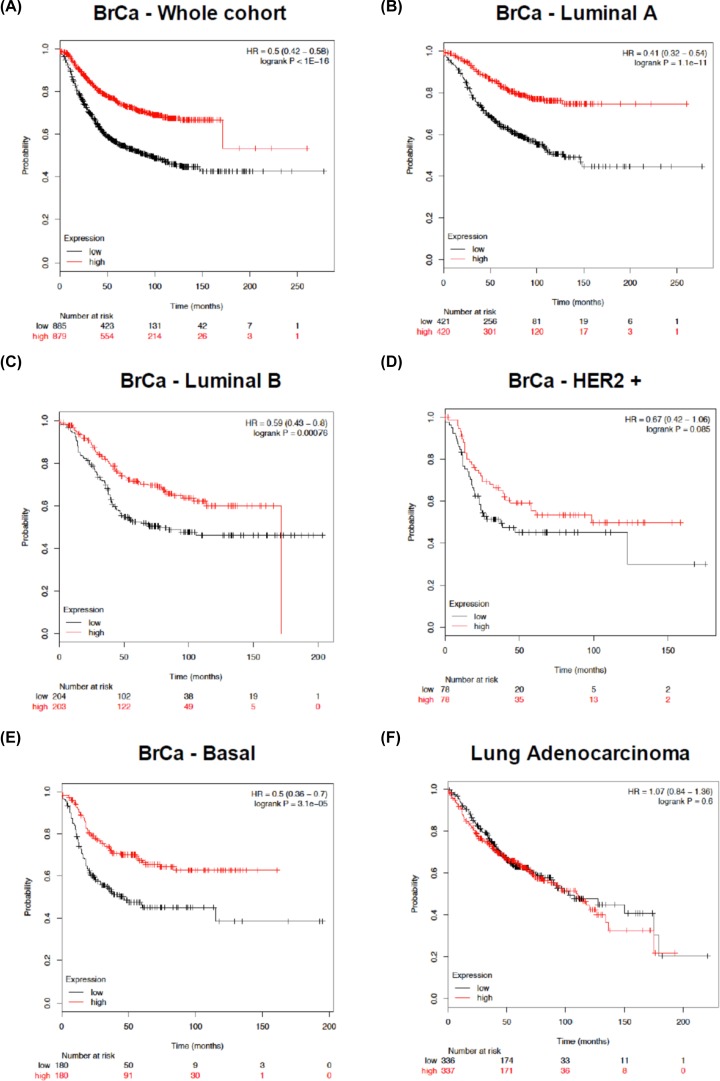
Prognostic potential of WDFY2 expression in cancer Kaplan–Meier curves of breast cancer (**A**–**E**) as well as LUAD (**F**) patients according to *WDFY2* expression levels using the KM Plotter online tool. RFS of each patient was plotted against the time of follow-up (25 years, expressed in months). Patients with high or low expression levels of *WDFY2* were presented with red and black lines, respectively. Within breast cancer patients, the entire cohort or the Luminal A, the Luminal B, the HER2 positive, and the Basal subgroups were specifically analysed. HR, significant *P*-values (logrank P) and numerosity are indicated within each panel.

Lastly, the prognostic potential of *WDFY2* expression in a large cohort of breast cancer patients was evaluated. Notably, higher levels of *WDFY2* appear to be associated with a remarkably better prognosis in terms of RFS (Figure 3A). By grouping the patients’ data according to the subtype classification (Luminal A, Luminal B, HER2+ and Basal) the prognostic value of *WDFY2* expression is confirmed for all the subgroups except the HER2+ class, with the highest significance for the Luminal A patients (Figure 3B–E). This observation is not confirmed for LUAD, as patients with low or high expression of *WDFY2* show the same prognosis (Figure 3F).

Taken collectively, despite the heterogeneous nature of these patients’ data, the results suggest a role for *WDFY2* as a tumour suppressor gene and the possible assessment of its expression level as a new prognostic biomarker.

### Placing *WDFY2* in the p63 network of limb development: identification of a reciprocal balanced translocation involving the *WDFY2* locus in a patient affected by split-hand/foot malformation with long-bone deficiency

**Figure 4:**
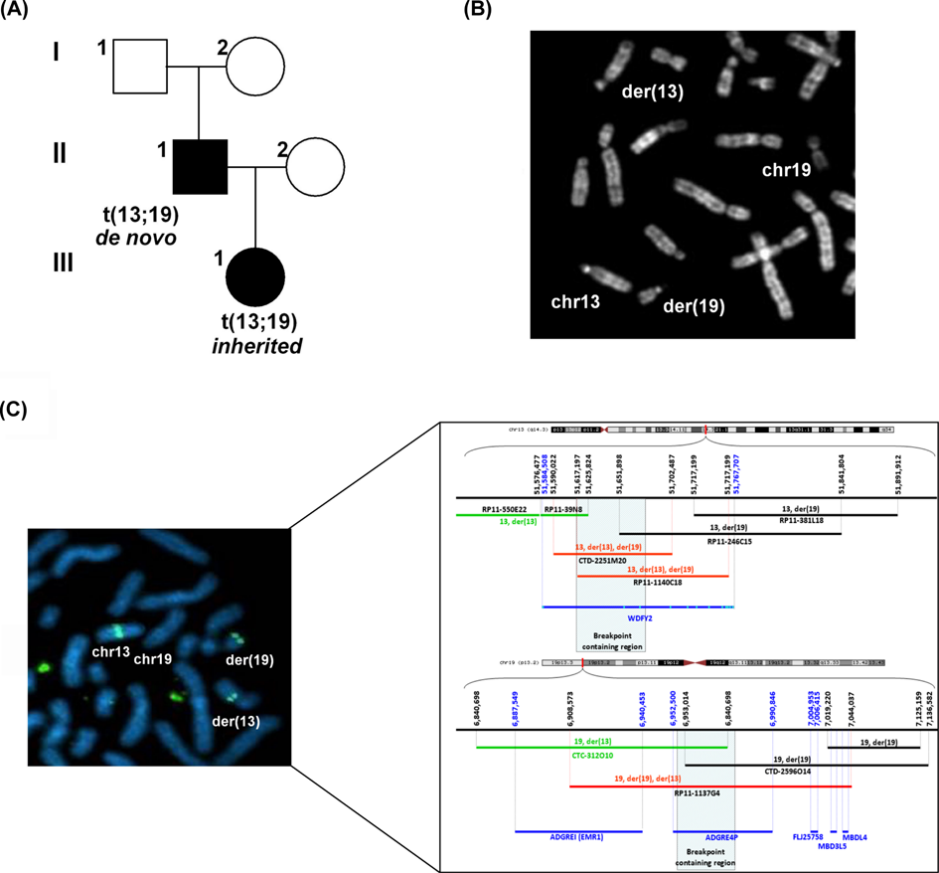
Identification of a chromosomal translocation involving *WDFY2* locus in a patient affected by a rare congenital limb anomaly (**A**) Pedigree showing the segregation of the t(13;19)(q14.3;p13.2) translocation. (**B**) Partial father’s metaphase by QFQ-banding showing the presence of the reciprocal balanced t(13;19)(q14.3;p13.2) translocation. Both normal and derivative chromosomes are indicated. (**C**) FISH analysis from father’s metaphase showing hybridisation with the CTD-2251M20 BAC probe spanning the breakpoint on chromosome 13. BAC clones relevant for the breakpoint definition are shown for chromosome 13 and 19. The breakpoint was mapped on chromosome 13 in the genomic region containing the *WDFY2* gene and on chromosome 19 in a region spanning the *ADGRE4P* pseudogene. Light blue lines in *WDFY2* gene indicate the position of the 12 coding exons.

A patient with clinical features suggestive of split-hand/foot malformation with long-bone deficiency (SHFLD) was identified in the present study. SHFLD is a rare and congenital condition that is characterised by split-hand/foot malformation, tibial aplasia or hypoplasia and sometimes, by malformations affecting other long bones. Karyotype analysis of the proposita revealed the presence of a reciprocal balanced translocation t(13;19)(q14.3;p13.2) that was also detected in the father but not in the mother (Figure 4A,B). The translocation was not found in the healthy parents of the father and thus considered *de novo* in this latter patient.

Interestingly, by FISH analysis with selected BAC clones covering the region spanning the translocation, the breakpoint was mapped on chromosome 13 in the genomic region containing the *WDFY2* gene (approximately 32 kb downstream of the first coding exon); the breakpoint on chromosome 19 was confined in a region spanning the *ADGRE4P* pseudogene (approximately 20 kb upstream of the first exon) (Figure 4C) that belongs to the EGF-TM7 receptor gene family. The results clearly indicate an interruption of the *WDFY2* gene unit due to the separation of the 5′ sequences with the corresponding promoter region and other regulatory elements from the downstream coding exons. Concerning the *ADGRE4P* pseudogene, the breakpoint containing region also interrupts this gene unit but data from literature about the functional relevance of this pseudogene are poor, being also unclear whether it is translated or not into a protein.

## Discussion

The p63 protein plays a key role in epithelial morphogenesis and cancer progression by encoding potentially ten different isoforms through the use of alternative promoters and splicing mechanisms [50]. p63 isoforms act mainly as transcription factors by binding to sequence-specific REs similar to those of p53 [15]. Several experimental approaches have been used to identify downstream p63 effectors, including microarrays, ChIP-based methods and ChIP-on-chip; results from these studies allowed the identification of the mechanisms by which p63 controls different molecular signalling involved in development and tumour formation, progression and metastasis [51].

By using ChIP-on-chip experiments, the *WDFY2* gene encoding a poorly investigated endosomal protein was identified amongst the 181 p63-bound loci in human immortalised keratinocyte HaCaT cells [19]. However, the authors confirmed the p63-dependent regulation of *WDFY2* exclusively by: (1) *in vivo* p63 binding in primary keratinocytes and (2) expression analysis in differentiating HaCaT, and in U2OS cells overexpressing ΔNp63α isoform.

Here, we demonstrated the *WDFY2* responsiveness to p63 transcription factor firstly by the identification and the functional characterisation of the p63-dependent regulatory elements. The search for p63 REs was based on TFBIND bioinformatics tool that, in accordance with other available online resources, allows the identification of p53 REs [52,53]. Currently, no specific software for searching for p63 (or p73) REs has been developed. This is in part due to the fact that p63 is able to recognise REs whose consensus sequence is highly similar to that of p53 REs, although p63 can display distinct requirements with respect to p53 for DNA sequence-specific binding and transactivation [16,54–56]. For example, contrary to p53, p63 was shown to preferentially activate sequences with a G in the 5th and 10th positions within the core of the RE, with specific C mismatches at the 11th and 16th positions contributing as well [16].

Since we previously observed that p63 isoforms are inactive on REs composed by a half-site and that p63 transactivation ability is affected by the presence of a spacer within the RE [17], we focused our analysis on canonical REs (i.e., 20 bps without a spacer) located between 10 kb upstream and 15 kb downstream of the TSS of human *WDFY2* gene. The TFBIND software identified seven REs (Table 1) that were analysed using a yeast-based functional assay [57] (and the references therein). Notably, all identified REs do not contain the previously described requirement for the p63-specific transactivation with exception of the promoter −0.5 RE that is characterised by the presence of a C at 16th position.

p63 isoforms are able to efficiently transactivate from a promoter and an intronic RE located at approximately 3 kb and 12 kb upstream and downstream of the *WDFY2* TSS, respectively (Figure 1). The potential of a shared regulation at the highly responsive intronic RE by all p53 family members (i.e., p53, p63 and p73) is also evident (Figure 1 and Supplementary Figure S1). Interestingly, p53 was frequently found bound to the region containing this RE in different cellular contexts and experimental conditions [58]. Moreover, by comparing p63 ChIP-seq data in actively proliferating primary human foreskin keratinocytes with pooled P53 ChIP-seq data following DNA damage from SaOs-2 cells (expressing exogenous p53) and IMR90 normal lung fibroblasts, McDade and colleagues [59] identified *WDFY2* as a potential common target gene, in keeping with our observations.

We also observed in yeast a higher transactivation potential of TA-P63β and TA-P63γ isoforms with respect to TA-P63α; on the contrary within ΔNp63 isoforms, ΔNp63α results to be the most transactivating one (Supplementary Figure 1).

Since the extent of p63 transactivation of the *WDFY2* gene may be due to the sum of the contribution of each functional RE, we analysed a promoter fragment of *WDFY2* locus, confirming the highest transactivation ability of the less expressed TAp63β and γ variants; with regard to ΔNp63 isoforms, ΔNp63β shows the maximal efficiency (Figure 2 and Supplementary Figure S2). These results are in agreement with previous data that revealed a high activity associated with ΔNp63β isoform in mammalian cell with respect to yeast context [17]. Moreover, the promoter analysis indicated a contribution of the −3.3 and −0.5 REs in transactivation potential especially of ΔNp63β, TAp63β and TAp63γ isoforms and a slight increase (∼1.5-fold) in transactivation potential of all TAp63 isoforms in the presence of +12.5 RE (Figure 2). A potential regulation of *WDFY2* promoter by the p53 protein (Supplementary Figure 2) is also evident.

As a second functional assay in a mammalian cell-based context, the endogenous expression levels of *WDFY2* were analysed. We identified an up-regulation of *WDFY2* mRNA level after the transfection of each P63 isoform, being the TA-P63 isoforms the most effective (two- to three-folds) (Figure 2). Moreover, in keeping with previous results and data from the literature [58,60], p53 protein is able to induce the *WDFY2* target about four-fold (Figure 2). Altogether, the results despite a weak induction of WDFY2 at protein level (Figure 2), confirmed the p63 responsiveness of the *WDFY2* gene.

Recently, a new role of *WDFY2* in cancer regulation is emerging; in fact, a *CDKN2D-WDFY2* fusion transcript encoding a shorter WDFY2 protein was frequently found in ovarian cancer samples and associated with a deregulated expression of members of the PI-3K/AKT pathway [61]. In prostate tumour tissues, a *WDFY2* down-regulation has been highlighted and correlated with advanced stage, increased metastasis and poor prognosis in patients, being AKT signalling modulation the mechanism adopted by WDFY2 protein to inhibit cancer progression and metastasis [62]. Lastly, WDFY2 was also shown to interact with endosomal Liver Kinase B1 (LKB1) protein and to participate in controlling its activity on the regulation of epithelial polarity and architecture; interestingly, the lack of this interaction endows LKB1 with tumour-promoting activity [63]. We thus decided to evaluate *WDFY2* genetic alterations, expression and prognostic significance using cancer patients’ data from different portals (cBioPortal, TCGA Consortium, GEPIA and KM Plotter) (Figure 3 and Supplementary Figures S3 and S4), consistently supporting this newly identified role of *WDFY2*.

We also placed the *WDFY2* gene in the p63-dependent network involved in limbs development. In fact, we report the recurrence of a reciprocal balanced translocation t(13;19)(q14.3; p13.2) in two related patients with a clinical picture suggestive of a diagnosis of SHFLD. The analysis of the translocation revealed the interruption of the *WDFY2* gene on chromosome 13, while on chromosome 19 the breakpoint was mapped in the *ADGRE4P* pseudogene (Figure 4), which is thought to play a role in leucocyte adhesion and migration [64]. The genetic aetiology of SHFLD is highly heterogeneous and although so far three loci have been mapped (Supplementary Table S4), no candidate genes were finally identified. Only for the SHFLD3 condition, duplication of the region spanning the *BHLH9* gene, has been described [65–67]. However, the CGH-Array analysis as well as the DNA sequencing of the *TP63* gene we performed did not reveal any additional alterations in the father of the proposita that harbours the *de novo* translocation t(13;19). This result, along with previous data in this manuscript, suggest *WDFY2* as a possible susceptibility gene in our patients to be placed in the p63 network of limb development. Firstly, it has been identified as an effector of *TP63* gene ([19] and present work) whose mutations have been recurrently reported in association with both isolated and syndromic split-hand/foot malformation [8,68]. Secondly, as revealed by RNA-seq expression data from GTEx (Release V6) [69], *WDFY2* expression is skin-specific (highest median expression: 13.56 RPKM in Skin - Sun Exposed) in contrast with the *ADGRE4P* pseudogene that is characterised by low levels in many tissues, being mainly expressed in the spleen (highest median expression: 5.36 RPKM). Lastly, the *in silico* analysis of human *ADGRE4P* genomic organisation did not reveal the presence of possible p63 REs. Then, we can suppose that alteration of *WDFY2* gene dosage due to the disruption of the locus in our patients might affect the PI-3K/AKT signalling, with which WDFY2 has been associated [61,62,70].

Interestingly, the PI-3K/AKT pathway is also involved in ectodermal organ development. Indeed, it has been shown that signalling induced by thalidomide, a drug known to induce congenital defects, comprising limb defects, stabilises PTEN and consequently suppresses the AKT pathway with the corresponding activation of apoptotic mechanisms in the limb bud during embryonic development [71]. Moreover, mice expressing a permanently activated Akt1 develop alterations in ectodermal organs that are similar to defects present in mice resembling human ectodermal dysplasia syndromes [72]. Lastly, AKT was also shown to interact with and to phosphorylate the homoeodomain transcription factor DLX5 [73], whose mutations have been linked to deficiencies in craniofacial and limb development in higher eukaryotes, including Split Hand and Foot Malformation-1 (SHFM-1) in humans [74]. All these observations lead us to further justify the role of p63 protein in regulating *WDFY2* expression, thus identifying the *WDFY2* gene as a p63 target potentially involved in the clinical manifestation of split hand/foot malformation.

In conclusion, this work confirmed the p63-dependent regulation of the endosomal WDFY2 protein, providing new clues for deciphering the downstream molecular pathways that are implicated in cancer regulation and limb development. *WDFY2* modulation appears to be also p53-dependent. Interestingly, recent studies have highlighted additional novel functions of p53 protein that include the down-regulation of the two central cell-growth pathways IGF/AKT-1 and mTOR and the up-regulation of the activities of the endosomal compartment [75], contributing to reduce cell growth and division. Therefore, the regulation of *WDFY2* might represent one of the molecular signalling by which also p63 protein can control tumorigenesis by inhibiting the AKT pathway.

## Supplementary Material

Supplementary Figures S1-S4 and Tables S1-S4

## Abbreviations

ChIP: chromatin immunoprecipitation
ER+: oestrogen receptor positive
GEPIA: gene expression profiling interactive analysis
HNSCC: head and neck squamous cell carcinoma
HR: hazard ratio
LKB1: liver kinase B1
LUAD: lung adenocarcinoma
PI3P: phosphatidyl-inositol 3-phosphate
RE: response element
RFS: relapse-free survival
RPKM: reads per kilobase per million mapped reads
SHFLD: Split-hand/foot malformation with long-bone deficiency
TCGA: The Cancer Genome Atlas
TNBC: triple-negative breast cancer
TSS: transcription start site
WDFY2: *WD* repeat and *FY*VE domain containing *2*
