# Supplementary material for: P63 modulates the expression of the WDFY2 gene which is implicated in cancer regulation and limb development
Source: Biosci Rep. 2019 Dec 13;39(12):BSR20192114. doi: 10.1042/BSR20192114 (PMC6914664; doi:10.1042/BSR20192114)
Supplement: Supplementary Figures S1-S4 and Tables S1-S4 [file BSR-2019-2114_supp.pdf]

Supplementary Materials and Methods

Cloning of yeast ΔNp63γ and TAp63γ isoforms expression vectors

Unexpectedly, by sequencing the original pCDNA3 plasmids, we highlighted a C-terminal deletion of 12 bps in *TP63γ* coding sequence, as described by Klein and co-workers [1]. Therefore, in order to clone the ΔNp63γ and TAp63γ isoforms we designed two PCR primers encompassing the p63 coding sequence from nucleotide 1140 to nucleotide 1170 downstream the C-terminal deletion (Supplementary Table 1). Forward primer was paired with a γ-Cter primer having a 5' homology to a XhoI/NotI (NEB, Ipswich, Massachusetts, USA) double digested yeast expression vector (pTS-based) and a 3' homology to *TP63γ* cDNA (Supplementary Table 1) in a PCR reaction (PCR A) with the above described pCDNA3 plasmids as template. Reverse primer was paired with ΔN-Nter or TA-Nter primer (Supplementary Table 1) [both having a 5' homology to the XhoI/NotI double digested yeast expression vector (pTS-based) and a 3' homology to ΔN or TA*TP63* cDNA] in a PCR reaction with pCDNA3.1 ΔN or TAp63α plasmid as template (PCR B and C, respectively). The PCR conditions were used as described in Marengo et al. [2]. Unpurified mixtures of PCR products (A+B or A+C) were then transformed in yIG397 yeast strain together with the XhoI/NotI digested pTS-based vector, exploiting the sequence homology at the end of the fragments (Gap Repair Assay) [3]. Plasmid DNA was recovered from yeast colonies, expanded in *E. coli*, and checked by restriction enzymes digestion; the presence of the correct ΔNp63γ and TAp63γ coding sequence was verified by DNA sequencing (BMR Genomics, Padua, Italy). Yeast vectors expressing ΔNp63γ and TAp63γ isoform under the inducible *GAL1,10* promoter (pTSG-based) were constructed as previously described [4].

Collection of clinical cases

The proposita is a 1-year-old girl born at term by cesarian section from non-consanguineous parents. A threatened miscarriage in the first trimester of pregnancy was referred; exposure to

drugs, smoke, alcohol or infections during pregnancy were denied. Ultrasonography at 20 weeks of pregnancy revealed the presence of a right clubfoot. At birth, her weight was 4.150 kg (90th-95th percentile), and her length was 50 cm (50th-75th percentile); occipitofrontal circumference and Apgar score were not reported. Perinatal period and psychomotor development were normal.

At the time of consultation, at the age of 1 year, the proposita showed cleft of the right hand with complete cutaneous syndactyly of the 3^{d} and 4th finger; short and extremely hypoplasic right tibia with homolateral clubfoot was also evident. Skin, nails, and sweating were normal. At the physical examination, the father of the proposita was found to carry defects of the right-hand central rays, with cleft hand surgically corrected and absence of the 3^{d} finger with thinned 3^{d} metacarpal bone. Feet were normal. At the physical examination, the mother appeared to be normal; no recurrence of malformations, mental retardation or other conditions were referred in the families of both parents.

The central rays' defects of the hand were classified as "split hand malformation"; in the proposita, this malformation was associated with a marked tibia hypoplasia. The clinical picture suggested in the proposita a diagnosis of SHFLD (Split-hand/foot malformation with long-bone deficiency).

SUPPLEMENTARY TABLES

Supplementary Table 1. Sequences of the oligonucleotides used in the present study are reported, with the indication of their specific application.

Cloning of p63 γ isoforms

p63 1140-1170 forward: 5’-**aacacacatggtatccagatgacatccatc**-3’
p63 1140-1170 reverse: 5’-**gatggatgtcatctggataccatgtgtgtgtt**-3’
p63 γ-Cter: 5’-gacataactaattacatgatggtggcggccgctctagaactagtggatcc**ctatgggtacactgatcggtt**-3’
p63 ΔN-Nter: 5’-caagctataccaagcatacaatcaactatctcatatacagttaactcgag**atgttgtacctggaaaacaat**-3’
p63 TA-Nter: 5’-caagctataccaagcatacaatcaactatctcatatacagttaactcgag**atgtcccagagcacacagaca**-3’

Generation and mutagenesis of the mammalian reporter constructs
Pr3300 WDProm-3F: 5’-ACTTGTGCTGGTCACAGCA-3’
Pr3300 WDProm-2R: 5’-ATCTGGTATCCCAATGCGCG-3’

+12.5 RE WD-F: 5’-GGATCCTACGGAGAAGAAGCGGTCC-3’
+12.5 RE WD-R: 5’-GGATCCTGACAATGATCTATACTTCCAC-3’

del-3.3 REas: 5’-GTCAAGATATTGGGCTGGAAATATCTTGTTTAGGAGCTTGAGTTTTAA-3’
del-3.3 REs: 5’-TTAAAACTCAAGCTCCTAAACAAGATATTTCCAGCCCAATATCTTGAC-3’

del-0.5 REas: 5’-GCCCAACTCGTTGGCGGGCTACCTGG-3’
del-0.5 REs: 5’-CCAGGTAGCCCGCCAACGAGTTGGGC-3’

Endogenous gene analysis

WDFY2 forward: 5’-GTGATCGTGCCCAAAGAGGA-3’
WDFY2 reverse: 5’-TCGTCACTCTGCTCTGATGC-3’

GAPDH forward: 5’-TCCAAAATCAAGTGGGGCGA-3’
GAPDH reverse: 5’-AGTAGAGGCAGGGATGATGT-3’

YWHAZ forward: 5’-ACTTTTGGTACTTTGTGGCTTCAA-3’
YWHAZ reverse: 5’-CCGCCAAGGGACAAACCAGTAT-3’

The nucleotides in **bold** correspond to the sequences that are complementary to *TP63* cDNA. The nucleotides underlined correspond to the sequence of BamHI restriction site that was added to the oligonucleotides for RE amplification.

Supplementary Table 2. Statistical analyses regarding the data showed in the main Figures.

-7.6 RE (Figure 1)	p53	ΔNp63α	ΔNp63β	TAp63α	TAp63β
p53	-	0.0002	0.0035	0.0018	0.0002
ΔNp63α	0.0002	-	0.0008	0.0001	0.0301
ΔNp63β	0.0035	0.0008	-	0.0001	0.0004
TAp63α	0.0018	0.0001	0.0001	-	<0.0001
TAp63β	0.0002	0.0301	0.0004	<0.0001	-

-3.3 RE (Figure 1)	p53	ΔNp63α	ΔNp63β	TAp63α	TAp63β
p53	-	NS	<0.0001	<0.0001	0.0003
ΔNp63α	NS	-	<0.0001	<0.0001	0.0002
ΔNp63β	<0.0001	<0.0001	-	0.0002	<0.0001
TAp63α	<0.0001	<0.0001	0.0002	-	<0.0001
TAp63β	0.0003	0.0002	<0.0001	<0.0001	-

-0.5 RE (Figure 1)	p53	ΔNp63α	ΔNp63β	TAp63α	TAp63β
p53	-	0.0031	NS	0.0013	<0.0001
ΔNp63α	0.0031	-	0.0026	<0.0001	0.0006
ΔNp63β	NS	0.0026	-	<0.0001	<0.0001
TAp63α	0.0013	<0.0001	<0.0001	-	<0.0001
TAp63β	<0.0001	0.0006	<0.0001	<0.0001	-

+12.5 RE (Figure 1)	p53	ΔNp63α	ΔNp63β	TAp63α	TAp63β
p53	-	<0.0001	<0.0001	<0.0001	<0.0001
ΔNp63α	<0.0001	-	<0.0001	0.0002	<0.0001
ΔNp63β	<0.0001	<0.0001	-	<0.0001	<0.0001
TAp63α	<0.0001	0.0002	<0.0001	-	<0.0001
TAp63β	<0.0001	<0.0001	<0.0001	<0.0001	-

+14.5 RE (Figure 1)	p53	ΔNp63α	ΔNp63β	TAp63α	TAp63β
p53	-	0.0081	NS	<0.0001	<0.0022
ΔNp63α	0.0081	-	<0.0001	<0.0001	0.0101
ΔNp63β	NS	<0.0001	-	<0.0001	0.0006
TAp63α	<0.0001	<0.0001	<0.0001	-	<0.0001
TAp63β	0.0022	0.0101	0.0006	<0.0001	-

Figure 2B	p53	ΔNp63α	ΔNp63β	ΔNp63γ	TAp63α	TAp63β	TAp63γ
p53	-	0.0145	0.0323	0.0236	Not quite	NS	NS
ΔNp63α	0.0145	-	Not quite	NS	0.0409	0.0080	0.0034
ΔNp63β	0.0323	Not quite	-	NS	NS	0.0235	0.0061
ΔNp63γ	0.0236	NS	NS	-	NS	0.0108	0.0020
TAp63α	Not quite	0.0409	NS	NS	-	Not quite	0.0257
TAp63β	NS	0.0080	0.0235	0.0108	Not quite	-	NS
TAp63γ	NS	0.0034	0.0061	0.0020	0.0257	NS	-

P values reported in red are showed in the corresponding Figures.

Supplementary Table 3. BAC clones used for FISH analysis with the description of the chromosomal location, clone name, accession number and corresponding genomic sequence.

**Chromosome 13q**		**GRCh38/hg38 Assembly**
**Clone**	**Acc. N.**	**Position**
RP11-550E22	AL354820	chr13: 51,410,490-51,576,476
RP11-39N8	AL139183	chr13: 51,576,377-51,625,824
CTD-2251M20	AQ192444	chr13: 51,590,022-51,702,487
RP11-147H23	AL136525	chr13: 51,625,725-51,771,064
RP11-1140C18	AQ707170	chr13: 51,617,197-51,762,535
CTD-3118D17	AQ338427	chr13: 51,651,898-51,841,804
RP11-246C15	AQ490062	chr13: 51,672,939-51,844,524
CTD-3036L15	AQ104587	chr13: 51,661,931-51,768,487
RP11-381L18	AL138821	chr13: 51,939,968-51,981,637
**Chromosome 19p**		**GRCh38/hg38 Assembly**
**Clone**	**Acc. N.**	**Position**
CTC-312O10	AC020895	chr19: 6,840,698-6,980,598
RP11-1137G4	AC025278	chr19: 6,908,573-7,044,037
RP11-303B11	AC068390	chr19: 6,922,669-6,948,786
CTD-2596O14	AQ478079	chr19: 6,953,014-7,136,582
CTB-25J19	AC010606	chr19: 7,019,220-7,125,159

Supplementary Table 4. Loci and genes associated to Split-Hand/Foot malformation with Long Bone Deficiency. For each locus the corresponding references are also reported.

OMIM ID	Condition	Inheritance	*Locus*	Comments	Reference
119100	Split-Hand/Foot malformation with Long Bone Deficiency 1; SHFLD1	AD	1q42.2-q43	*Locus* identified by genome-wide linkage analysis, no gene candidate gene identified	[5], [6]
610685	Split-Hand/Foot malformation with Long Bone Deficiency 2; SHFLD2	AD	6q14.1	*Locus* identified by genome-wide linkage analysis, no gene candidate gene identified	[5], [6]
612576	Split-Hand/Foot malformation with Long Bone Deficiency 3; SHFLD3. Chromosome 17p13.3, Telomeric Duplication Syndrome	AD	17p13.3-p13.1	Identification of duplications of different size encompassing the *BHLHA9* gene	[7], [8]

Supplementary Figures Legends

Supplementary Figure 1. P73 and P63 isoforms transactivation from REs belonging to *WDFY2* gene by using a yeast-based reporter assay

A) Transactivation ability of p73 isoforms in yLFM-WDFY2 yeast strains containing the reporter gene under the regulation of a promoter (-7.6, -4.3, -3.3 and -0.5) or intron 1 (+12.5, +14.5 and +15.7) RE from the human *WDFY2* gene. The transactivation ability was determined and presented as in Figure 1. **B)** Transactivation ability of p63 isoforms in yLFM-WDFY2 (-3.3 RE) and yLFM-WDFY2 (+12.5 RE) yeast strains. The transactivation ability was determined and presented as in Figure 1 but by growing yeast cells for 8 hours in media containing 0.016% or 0.128% Galactose. **C)** Representative western blots showing the expression levels of p63 isoforms in yeast cell lysates derived from the functional assay (0.128% Galactose). PGK1 was used for normalization. Histogram showing the average level of p63 isoforms in yeast from three independent western blot experiments.

Supplementary Figure 2. Regulation of *WDFY2* expression by P63 isoforms and P53 in human cells

A) Representative western blot showing the level of p63 protein isoforms found in HCT116 *TP53*$^{-/-}$ cell lysates following transient co-trasfection with the indicated p63 expression vectors and promoter-derived reporter plasmids. β-actin was used for normalization. Histogram showing the average level of p63 isoforms expressed in HCT116 *TP53*$^{-/-}$ cells from three independent western blot experiments. **B)** Transactivation ability of p53 on reporter constructs Pr3300 and Pr3300 del-3.3/-0.5 REs in HCT116 *TP53*$^{-/-}$ human cells. Renilla luciferase was used to normalize for transfection efficiencies. Data are expressed as in Figure 2. **C)** A representative western blot showing the level of p53 protein found in HCT116 *TP53*$^{-/-}$ cell lysates following transient co-transfection of p53 expression vector with the indicated promoter-derived reporter plasmids. β-

Actin was used for normalization. -, empty vector.

Supplementary Figure 3. *WDFY2* alterations in cancer

A) *WDFY2* mutations (missense, truncating and other mutations) are shown according to the frequency (Y axis) and the position of the amino acid hit (X axis). WDFY2 functional domains are indicated as coloured boxes. **B, C)** Genetic events affecting *WDFY2* gene were analysed using the cBioPortal online tool (TCGA). Deletions, amplifications, mutations and fusions were presented as bars according to their specific frequency from the indicated collection cancer studies (indicated in the X axis). The cancer studies were ordered on the basis of the most frequent observed alteration (i.e., deletions in panel B and mutations or amplifications in panel C). The total number of patients is indicated in the brackets.

Supplementary Figure 4. Evaluation of *WDFY2* expression in cancer

A-D) Relative expression values (RPKM) of *WDFY2* using RNA-seq data (TCGA_Pancancer 12) from tumour (green circles) or normal matched tissues (light blue squares) of different origin (LUAD, Lung Adenocarcinoma; BRCA, Breast Cancer; RCC, Renal Clear cell Carcinoma; HNSCC, Head and Neck Squamous Cell Carcinoma). Numerosity of the samples is indicated on each panel (T = Tumour and N = Normal tissues). **E)** Relative expression values (RPKM) of *WDFY2* using an RNA-seq dataset from different subtypes of breast cancer patients. Shown are the medians and the interquartile ranges. * = $p < 0.01$ Student's T-test. **F)** A dot plot showing the relative expression values ($\log_2$ TPM+1) of *WDFY2* using the gene expression profiling interactive analysis database (GEPIA) from several cancer types (red dots) and matched controls (green dots). Medians are marked by a hyphen. Number of cases and controls are presented below the plot. For cancer type abbreviations see the GEPIA website (http://gepia.cancer-pku.cn/detail.php?gene=WDFY2); cancer type acronyms are coloured in green when *WDFY2* expression in tumours is significantly lower than in controls. Conversely, cancer type acronyms are

coloured in red.

REFERENCES

1. Klein, C., et al., *High thermostability and lack of cooperative DNA binding distinguish the p63 core domain from the homologous tumor suppressor p53.* J Biol Chem, 2001. **276**(40): p. 37390-401.
2. Marengo, B., et al., *Etoposide-resistance in a neuroblastoma model cell line is associated with 13q14.3 mono-allelic deletion and miRNA-15a/16-1 down-regulation.* Sci Rep, 2018. **8**(1): p. 13762.
3. Flaman, J.M., et al., *A simple p53 functional assay for screening cell lines, blood, and tumors.* Proc Natl Acad Sci U S A, 1995. **92**(9): p. 3963-7.
4. Monti, P., et al., *N-P63alpha and TA-P63alpha exhibit intrinsic differences in transactivation specificities that depend on distinct features of DNA target sites.* Oncotarget, 2014. **5**(8): p. 2116-30.
5. Naveed, M., et al., *Ectrodactyly with aplasia of long bones (OMIM; 119100) in a large inbred Arab family with an apparent autosomal dominant inheritance and reduced penetrance: clinical and genetic analysis.* Am J Med Genet A, 2006. **140**(13): p. 1440-6.
6. Naveed, M., et al., *Genomewide linkage scan for split-hand/foot malformation with long-bone deficiency in a large Arab family identifies two novel susceptibility loci on chromosomes 1q42.2-q43 and 6q14.1.* Am J Hum Genet, 2007. **80**(1): p. 105-11.
7. Klopocki, E., et al., *Duplications of BHLHA9 are associated with ectrodactyly and tibia hemimelia inherited in non-Mendelian fashion.* J Med Genet, 2012. **49**(2): p. 119-25.
8. Paththinige, C.S., et al., *Split hand/foot malformation with long bone deficiency associated with BHLHA9 gene duplication: a case report and review of literature.* BMC Med Genet, 2019. **20**(1): p. 108.

A

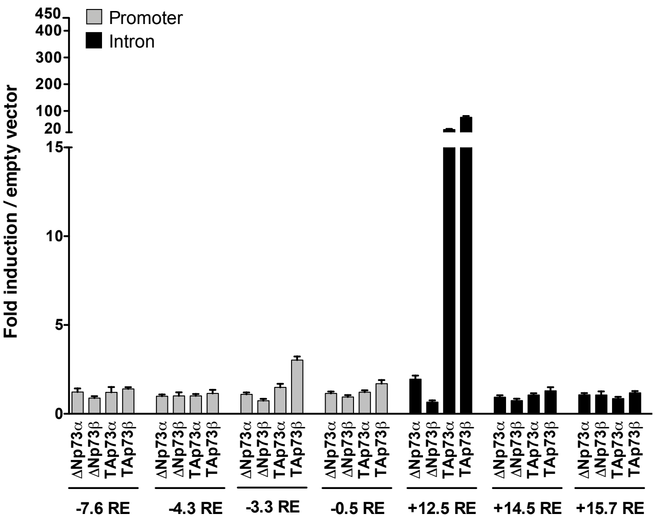

Promoter
Intron
Fold induction / empty vector
450
400
300
200
100
20
15
10
5
0
ΔNp73α
ΔNp73β
TAp73α
TAp73β
-7.6 RE
-4.3 RE
-3.3 RE
-0.5 RE
+12.5 RE
+14.5 RE
+15.7 RE

B

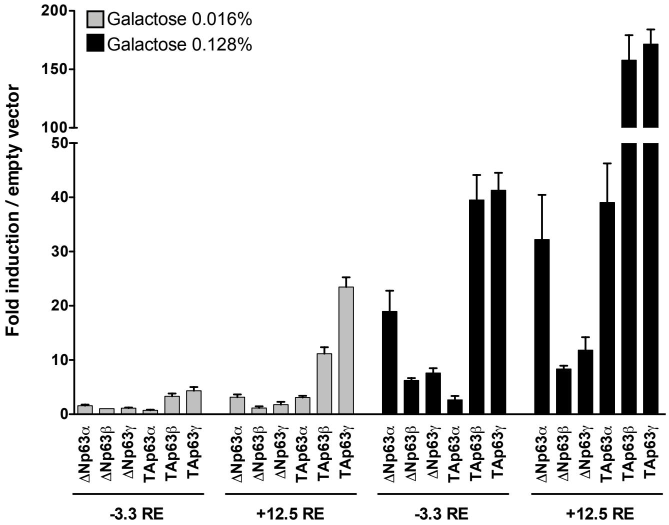

Galactose 0.016%
Galactose 0.128%
Fold induction / empty vector
200
150
100
50
40
30
20
10
0
ΔNp63α
ΔNp63β
ΔNp63γ
TAp63α
TAp63β
TAp63γ
-3.3 RE
+12.5 RE
-3.3 RE
+12.5 RE

C

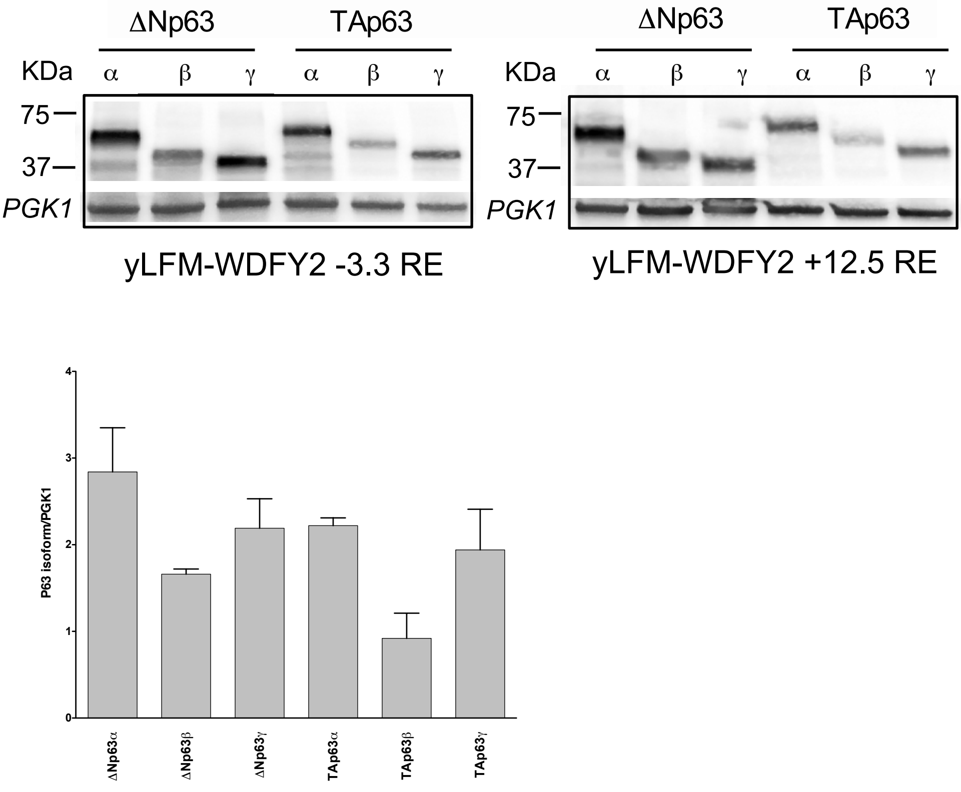

ΔNp63
TAp63
KDa
α
β
γ
75
37
PGK1
yLFM-WDFY2 -3.3 RE
yLFM-WDFY2 +12.5 RE
P63 isoform/PGK1
4
3
2
1
0
ΔNp63α
ΔNp63β
ΔNp63γ
TAp63α
TAp63β
TAp63γ

A

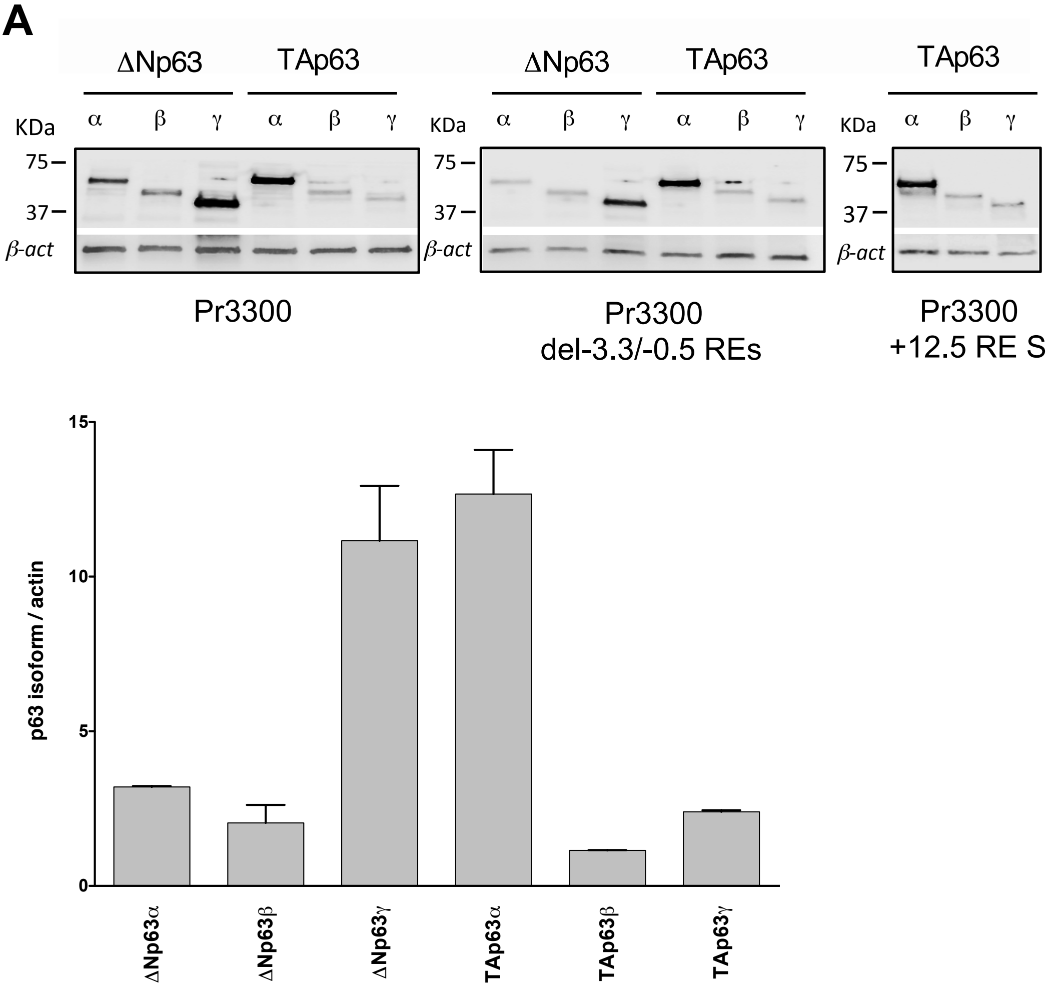
ΔNp63
TAp63
KDa
α
β
γ
75
37
β-act
Pr3300
Pr3300
del-3.3/-0.5 REs
Pr3300
+12.5 RE S
p63 isoform / actin
15
10
5
0
ΔNp63α
ΔNp63β
ΔNp63γ
TAp63α
TAp63β
TAp63γ

B

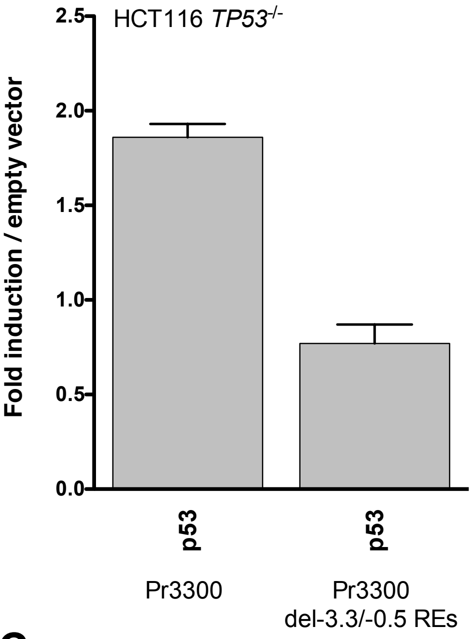
HCT116 TP53-/-
Fold induction / empty vector
2.5
2.0
1.5
1.0
0.5
0.0
p53
p53
Pr3300
Pr3300
del-3.3/-0.5 REs

C

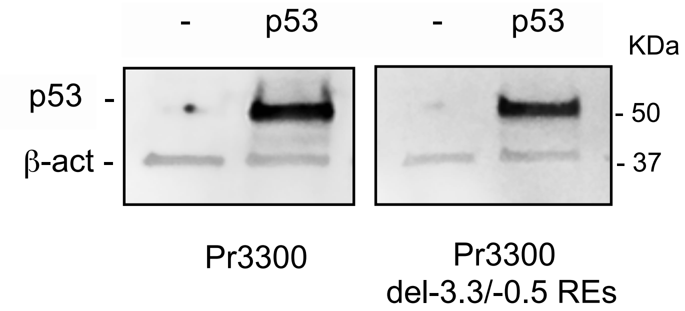
-
p53
-
p53
KDa
p53 -
β-act -
- 50
- 37
Pr3300
Pr3300
del-3.3/-0.5 REs

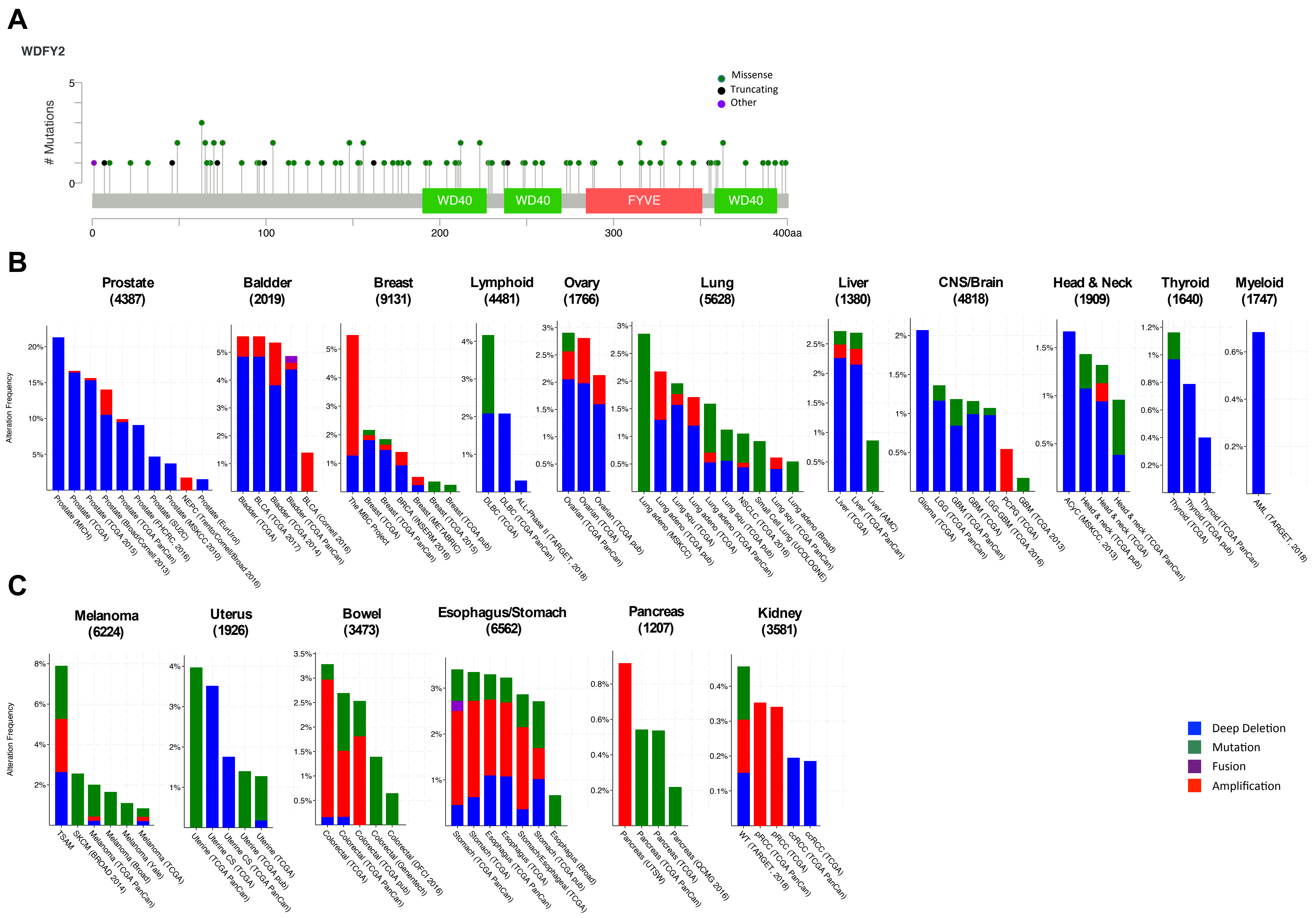
A
WDFY2
Mutations
5
0
Missense
Truncating
Other
WD40
WD40
FYVE
WD40
0
100
200
300
400aa
B
Prostate (4387)
Baldder (2019)
Breast (9131)
Lymphoid (4481)
Ovary (1766)
Lung (5628)
Liver (1380)
CNS/Brain (4818)
Head & Neck (1909)
Thyroid (1640)
Myeloid (1747)
Alteration Frequency
Prostate (MICH)
Prostate (TCGA)
Prostate (TCGA 2015)
Prostate (Broad/Cornell 2013)
Prostate (TCGA PanCan)
Prostate (FHCRC, 2016)
Prostate (SU2C)
Prostate (MSKCC 2010)
NEPC (Trento/Cornell/Broad 2016)
Prostate (EurUrol)
Bladder (TCGA)
BLCA (TCGA 2017)
Bladder (TCGA 2014)
Bladder (TCGA PanCan)
BLCA (Cornell 2016)
The MBC Project
Breast (TCGA)
Breast (TCGA PanCan)
BRCA (INSERM 2016)
Breast (METABRIC)
Breast (TCGA 2015)
Breast (TCGA pub)
DLBC (TCGA)
DLBC (TCGA PanCan)
ALL-Phase II (TARGET, 2018)
Ovarian (TCGA)
Ovarian (TCGA PanCan)
Ovarian (TCGA pub)
Lung adeno (MSKCC)
Lung adeno (TCGA pub)
Lung squ (TCGA)
Lung adeno (TCGA)
Lung adeno (TCGA PanCan)
Lung squ (TCGA pub)
NSCLC (TCGA 2016)
Small Cell Lung (UCOLOGNE)
Lung squ (TCGA PanCan)
Lung adeno (Broad)
Liver (TCGA)
Liver (TCGA PanCan)
Liver (AMC)
Glioma (TCGA)
LGG (TCGA PanCan)
GBM (TCGA PanCan)
GBM (TCGA)
LGG-GBM (TCGA 2016)
PCPG (TCGA)
GBM (TCGA 2013)
ACyC (MSKCC, 2013)
Head & neck (TCGA)
Head & neck (TCGA pub)
Head & neck (TCGA PanCan)
Thyroid (TCGA)
Thyroid (TCGA pub)
Thyroid (TCGA PanCan)
AML (TARGET, 2018)
C
Melanoma (6224)
Uterus (1926)
Bowel (3473)
Esophagus/Stomach (6562)
Pancreas (1207)
Kidney (3581)
Alteration Frequency
TSAM
SKCM (BROAD 2014)
Melanoma (TCGA PanCan)
Melanoma (Broad)
Melanoma (Yale)
Melanoma (TCGA)
Uterine (TCGA PanCan)
Uterine CS (TCGA)
Uterine CS (TCGA PanCan)
Uterine (TCGA pub)
Uterine (TCGA)
Colorectal (TCGA)
Colorectal (TCGA PanCan)
Colorectal (TCGA pub)
Colorectal (Genentech)
Colorectal (DFCI 2016)
Stomach (TCGA PanCan)
Stomach (TCGA)
Esophagus (TCGA PanCan)
Esophagus (TCGA)
Stomach/Esophageal (TCGA)
Stomach (TCGA pub)
Esophagus (Broad)
Pancreas (UTSW)
Pancreas (TCGA PanCan)
Pancreas (TCGA)
Pancreas (QCMG 2016)
WT (TARGET, 2018)
pRCC (TCGA PanCan)
pRCC (TCGA)
ccRCC (TCGA PanCan)
ccRCC (TCGA)
Deep Deletion
Mutation
Fusion
Amplification

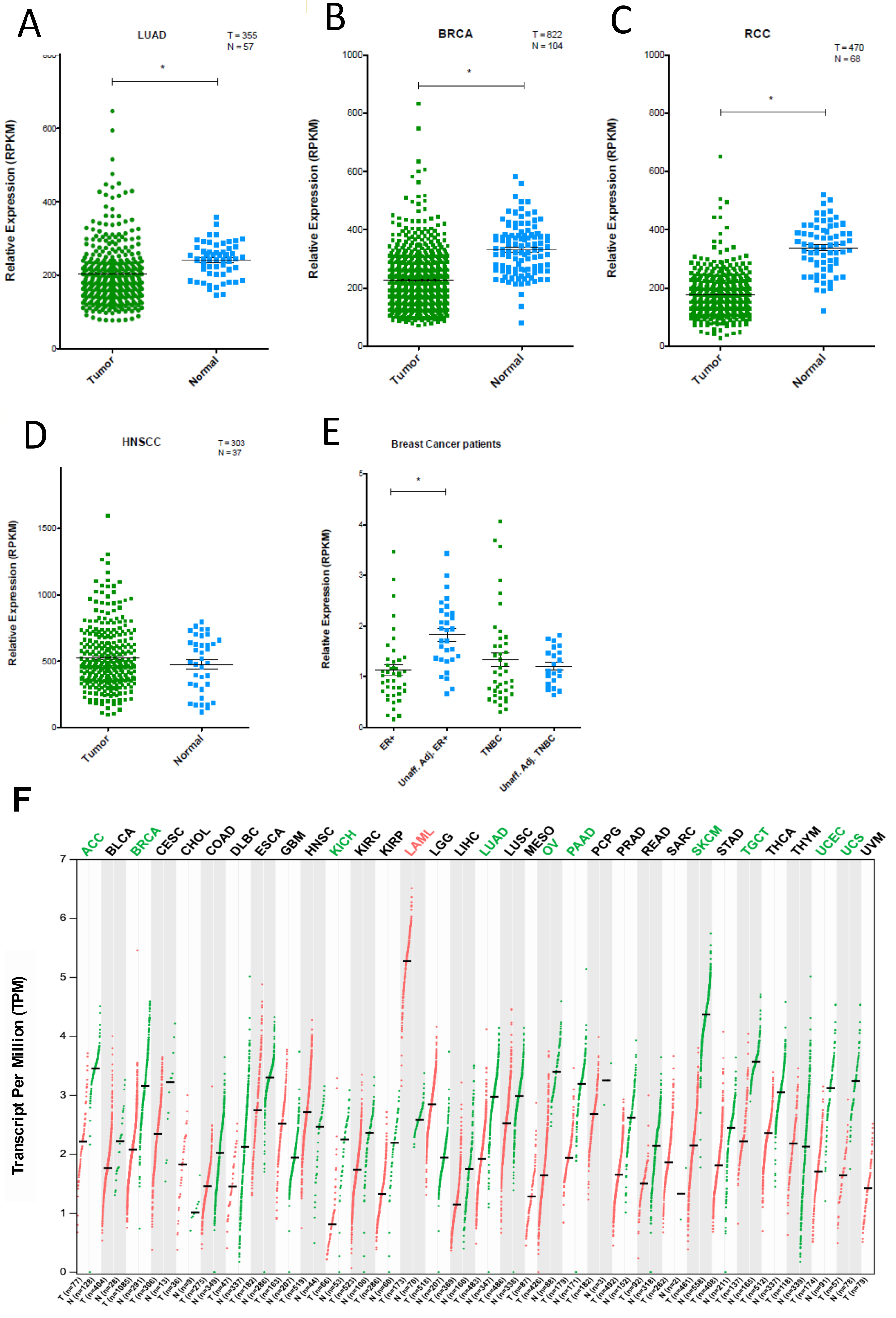
A
LUAD
T = 355
N = 57
B
BRCA
T = 822
N = 104
C
RCC
T = 470
N = 68
Relative Expression (RPKM)
Tumor
Normal
D
HNSCC
T = 303
N = 37
E
Breast Cancer patients
ER+
Unaff. Adj. ER+
TNBC
Unaff. Adj. TNBC
F
ACC
BLCA
BRCA
CESC
CHOL
COAD
DLBC
ESCA
GBM
HNSC
KICH
KIRC
KIRP
LAML
LGG
LIHC
LUAD
LUSC
MESO
OV
PAAD
PCPG
PRAD
READ
SARC
SKCM
STAD
TGCT
THCA
THYM
UCEC
UCS
UVM
Transcript Per Million (TPM)
